# Long-range correlations in alpha-band of electroencephalogram: a nonlinear embedding and detrended fluctuation analysis

**DOI:** 10.3389/fninf.2026.1823408

**Published:** 2026-05-20

**Authors:** Dasari Shivakumar, Cota Navin Gupta, Budhaditya Hazra

**Affiliations:** 1 Neural Engineering Lab, Department of Biosciences and Bioengineering, Indian Institute of Technology Guwahati, Assam, India; 2 Department of Civil Engineering, Indian Institute of Technology Guwahati, Assam, India

**Keywords:** detrended fluctuation analysis, electroencephalogram, isometric mapping, long-range temporal correlation, music, scaling exponent

## Abstract

Understanding the temporal organization of brain activity requires methods that capture scale-free dynamics while accounting for the high-dimensional, spatially correlated nature of the electroencephalogram (EEG) data. We propose a novel framework that integrates nonlinear manifold learning (Isometric mapping) with detrended fluctuation analysis (DFA) to quantify long-range temporal correlations (LRTC) in the alpha-band of EEG signals. We applied this framework to two music related EEG datasets, as music is known to evoke different emotions and synchronize brain activity. The first dataset was obtained during live Indian classical music (ICM) listening that included two ragas, Yaman and Puriya Dhanashree. EEG was recorded from 13 healthy volunteers (24 channels, sampled at 500 Hz). The second dataset is the Music BCI dataset (006-2015), which includes Jazz and Synth-pop musical clips, with EEG collected from 11 subjects (64 channels, downsampled to 200 Hz). The EEG data from both datasets were preprocessed, band-limited to 8–13 Hz, and segmented into non-overlapping 2-s windows. Alpha-band power was extracted from each channel to form the feature matrix used for embedding. For the ICM dataset, Isometric mapping (Isomap) produced a low-dimensional representation (*d* = 3), which we analyzed using two approaches: (i) a norm-based approach and (ii) a mean-based approach. For comparison, an equivalent PCA-based pipeline (*d* = 5) was implemented. The Isomap mean-based DFA yielded consistent scaling exponents (α) in the range of 0.66–0.70, with higher goodness-of-fit (*R*^2^) and narrower bootstrap confidence intervals than the norm-based approach. PCA produced similar trends but required more dimensions. Paired *t*-tests showed that the Isomap mean-based approach detected music-related changes more sensitively than PCA (Yaman *p* = 0.02; Puriya Dhanashree *p* = 0.008). Comparable results were also observed for the second Music BCI dataset, where Isomap achieved a compact representation with *d* = 5, compared to *d* = 8 for PCA. In this dataset as well, the mean-based DFA yielded α values in the range of 0.62–0.65 and higher goodness-of-fit. Overall, the results suggest that combining nonlinear manifold embeddings with mean-based DFA provides a compact and robust framework for characterizing scale-free temporal structure in EEG data.

## 1 Introduction

Electroencephalography (EEG) records the brain's electrical activity from the scalp, yielding complex and nonlinear signals. Its superior temporal resolution is crucial for capturing rapid brain dynamics (Niedermeyer and da Silva, 2005). Numerous studies have shown that neural activity shows long-range temporal correlations (LRTC) consistent with fractal organization (Banerjee et al., 2016; Linkenkaer-Hansen et al., 2001). Assessing such correlations is important to understand how the brain maintains memory of past events and adapts to external stimuli (Hardstone et al., 2012). For quantifying LRTCs in nonstationary time series data, a well-known technique known as detrended fluctuation analysis (DFA) is used (Peng et al., 1995, 1994; Pavlov et al., 2020). DFA technique has been widely applied to EEG and magnetoencephalography (MEG) data to reveal scale-free dynamics during rest, task performance, and sleep (Linkenkaer-Hansen et al., 2001; Hardstone et al., 2012). These findings suggest that DFA can capture fundamental aspects of neural temporal organization across conditions, making it a suitable tool to investigate the effects of external stimuli such as music.

EEG signals are inherently nonlinear and exhibit strong spatial correlations across channels, resulting in high-dimensional yet redundant representations of brain activity (Stam, 2005; Sanei and Chambers, 2013; Nunez and Srinivasan, 2006). Such redundancy suggests that the underlying neural dynamics may lie on a lower-dimensional manifold embedded within the sensor space, as observed in studies of large-scale brain dynamics and electrophysiological recordings (Andrzejak et al., 2001; Mehrkanoon et al., 2014). Previous studies have shown that neural activity can often be characterized by low-dimensional representations, reflecting coordinated dynamics across brain regions (Mehrkanoon et al., 2014; Yu et al., 2022). To address this high-dimensional redundancy, dimensionality reduction techniques are used to extract informative components while preserving underlying dynamics. Linear dimensionality reduction techniques such as principal component analysis (PCA) are commonly used to obtain lower-dimensional representations (Jolliffe, 2011). The PCA captures the variance along orthogonal directions in Euclidean space, but fails to capture nonlinear dependencies. Whereas manifold learning methods are used to preserve the intrinsic geometry of the data by approximating geodesic distances on the underlying manifold. The Isometric mapping (Isomap) technique (Tenenbaum et al., 2000) is one of the nonlinear dimensionality reduction methods, which combines local nearest-neighbor graphs with multidimensional scaling (MDS) to recover global nonlinear structure. Applications of Isomap and related algorithms have demonstrated advantages in uncovering low-dimensional embeddings of complex biological and neural data (Saul and Roweis, 2003; Ashraf et al., 2023; Mitchell-Heggs et al., 2023; Anuragi et al., 2024). This motivates the use of nonlinear dimensionality reduction as a more robust representation of EEG dynamics compared to linear projections.

Beyond classical dimensionality reduction techniques, recent advances in deep learning have enabled powerful data-driven representation learning frameworks for EEG analysis. BCINetV1 (Aziz et al., 2025) employs a convolutional attention-based architecture that integrates temporal and spectral feature extraction to capture both local and global dependencies in non-stationary EEG signals. Similarly, large-scale models such as the Large Brain Model (LaBraM) (Jiang et al., 2024) utilize transformer-based architectures trained on multiple EEG datasets to learn generalized representations across diverse tasks. In addition, EEGPT (Wang et al., 2024) introduces a pretrained transformer model based on self-supervised learning, incorporating spatio-temporal representation alignment and reconstruction objectives to extract robust EEG features. While these approaches have demonstrated strong performance in EEG decoding and classification tasks, they primarily focus on learning hierarchical representations optimized for predictive performance. In contrast, the present work adopts a complementary perspective by focusing on interpretable and mathematically grounded analysis of EEG dynamics.

Despite the extensive use of DFA to quantify scale-free properties in EEG (Banerjee et al., 2016; Linkenkaer-Hansen et al., 2001; Hardstone et al., 2012; Karkare et al., 2009; Grubov et al., 2025) and the growing application of dimensionality reduction techniques (Jolliffe, 2011; Tenenbaum et al., 2000; Saul and Roweis, 2003), relatively little work has integrated nonlinear manifold embeddings with fractal analysis. Most existing studies apply DFA directly to individual EEG channels, yielding interpretations restricted to those cortical regions (Banerjee et al., 2016; Pavlov et al., 2018) only. These approaches overlook broad synchrony across channels and fail to provide a global representation of brain dynamics. These limitations motivate the need for the development of a framework that combines nonlinear dimensionality reduction techniques with DFA to study long-range temporal correlations in EEG at a global scale. To the best of our knowledge, no prior literature works have explored this direction.

In contrast to traditional approaches where DFA is applied directly to EEG channels, we first reduce the dimension of the data using the Isomap technique. This approach assumes that the EEG data lie on an intrinsically curved, low-dimensional manifold rather than in the ambient sensor space. Isomap preserves this geodesic structure more effectively than linear methods. By applying DFA to the lower-dimensional embeddings obtained through Isomap, we characterize LRTCs, which more closely reflect the underlying brain dynamics. This integration of nonlinear dimensionality reduction with DFA provides a novel framework to uncover the hidden temporal structures in EEG activity. The main contributions of this work are as follows: *Firstly*, we propose a framework that integrates Isomap with detrended fluctuation analysis (DFA) for the study of EEG dynamics. *Secondly*, within this framework, we opted for two approaches to perform DFA on low-dimensional embeddings: a norm-based approach and a mean-based approach. *Thirdly*, we compare the proposed framework with the traditional linear dimensionality reduction technique using PCA.

## 2 Dataset and subjects

### 2.1 Subjects

For this study, 13 healthy individuals were recruited, including 11 males and two females, with a mean age of 30.54 ± 9.42 years. The demographic details of the participants are provided in Table 1. None of the participants had a history of neurological disorders and hearing loss. Before data collection, a clear demonstration of the experimental procedure was given to each subject. EEG data was recorded using a 24-channel cap, with electrodes placed according to the standard international 10–20 system (Jasper, 1958). The EEG data were acquired with the CameraEEG application using a mobile phone (Hazarika et al., 2023). To ensure signal quality, electrodes were cleaned with alcohol, conductive gel was applied, and impedances were maintained between 10 and 20 kΩ. Permission was obtained from the institute's ethics committee at IIT Guwahati for collecting data from human participants.

**Table 1:** Demographic data of the volunteers participated in the study.

Subjects	Gender	Age (years)	Prior music knowledge
Subject 1	M	21	No
Subject 2	M	45	No
Subject 3	M	26	No
Subject 4	F	25	No
Subject 5	M	31	No
Subject 6	M	28	No
Subject 7	M	28	Yes
Subject 8	M	41	Yes
Subject 9	M	27	No
Subject 10	M	25	No
Subject 11	F	52	Yes
Subject 12	M	25	No
Subject 13	M	23	Yes

### 2.2 Dataset used

**Figure 1:**
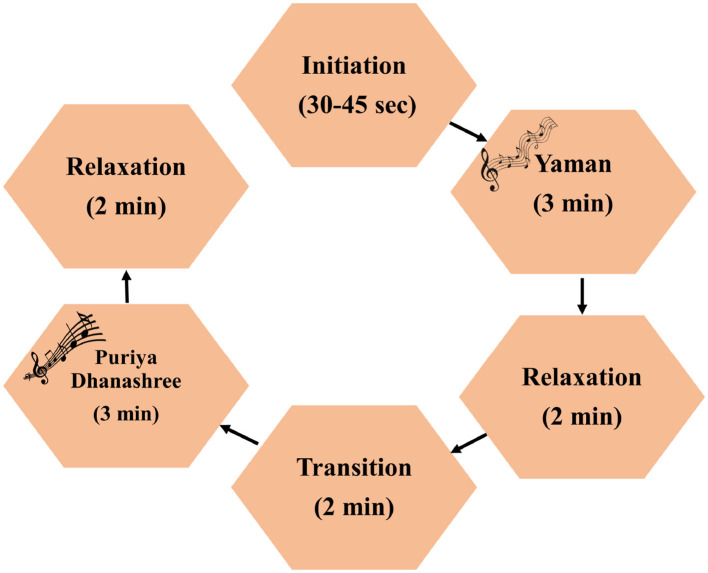
Live musical paradigm used for data collection for this study. Consists of two ragas: Yaman and Puriya Dhanashree, and relaxation conditions.

We used an EEG dataset from a music paradigm employed in the studies (Panda et al., 2024, 2023). The EEG data were obtained while subjects listened to a live vocal musical paradigm. It consisted of two ragas: Yaman and Puriya Dhanashree (PD). Data were collected on different days depending on the availability of participants. The musical paradigm is shown in Figure 1. It begins with an initiation phase, which lasts 30–45 s, and then enters Raga Yaman. This is followed by a relaxation period and then a transition phase. After the transition phase, raga Puriya Dhanashree (PD) is played, followed by another relaxation period. No music was played during either of the relaxation periods, although the EEG data were recorded. Each raga (Yaman and PD) lasted 3 min, and both relaxation conditions were 2 min long. The transition phase comprised 1 min of Raga Yaman followed by 1 min of Raga PD. Since it was a live musical paradigm, the timestamps of music and relaxation periods varied across subjects.

### 2.3 Preprocessing

EEG data were recorded while participants listened to the musical paradigm with their eyes closed. During the relaxation periods, participants were allowed to open their eyes and observe the surroundings. The data were sampled at a frequency of 500 Hz. Faulty channels were excluded from the study, and then artifact correction was performed using the multichannel wiener filter (MWF) toolbox in MATLAB (Somers et al., 2018). It removes eye blinks, muscle, and movement artifacts. Then, the EEG data were normalized using z-score normalization. Finally, the EEG signals were band-pass filtered in the alpha band (8–13 Hz). All subsequent analyses in this study were conducted on the alpha band (Panda et al., 2024).

## 3 Methodology

This section outlines the methodology adopted for applying detrended fluctuation analysis (DFA) on the low-dimensional manifold obtained using the isometric mapping (Isomap) technique. The pipeline consists of three main steps: (i). Extraction of alpha band (8–13 Hz) power, (ii). Dimensionality reduction of EEG data using Isomap, and (iii). Application of the DFA algorithm to the low-dimensional embedding.

### 3.1 Alpha-band (8–13 Hz) power

EEG data were acquired using a 24-channel cap at a sampling frequency (*fs*) of 500 Hz. The recordings are represented by a matrix *X*∈ℝ^*N*×*T*^, where *N* is the number of channels (*N* = 24) and *T* the number of time points. Then, the EEG signal was segmented into *n* non-overlapping 2-s windows. The number of windows *n* was computed using the ‘floor' function in MATLAB. This ensures that only complete windows are considered. Any remaining samples at the end of the recording that do not form a full window were discarded and not included in the analysis.


$$\begin{array}{c} n=\frac{T}{L} \end{array}$$ (1)

In Equation 1, the window length (*L* = 2·*fs*) of 2 s was chosen to provide sufficient frequency resolution (0.5 Hz) for capturing the alpha band of the EEG signal, consistent with prior EEG studies (Klimesch, 1999; Sanei and Chambers, 2013). Although EEG signals may not be strictly stationary within these windows, detrended fluctuation analysis (DFA) is specifically designed to handle nonstationary time series. The detrending step in DFA removes local trends within each segment, thereby reducing the influence of nonstationarities on the estimation of the scaling exponents (Linkenkaer-Hansen et al., 2001; Peng et al., 1994; Kantelhardt et al., 2001). For each 2-s window, the alpha-band power was computed for every channel using MATLAB's function ‘bandpower'. This function estimates the power spectral density (PSD) using Welch's method (Fast Fourier Transform-based) and then integrates the power within the alpha-band (8–13 Hz). These values were stored in a matrix $X_{\alpha }\in {\mathbb{R}}^{n\times N}$, where *n* is the number of windows and *N* the number of channels (*N* = 24). For subjects with one or two faulty channels removed, *N* = 24 was reduced to 23 or 22, respectively.

### 3.2 Dimensionality reduction of EEG data using Isomap

To reduce the dimensionality of the EEG data matrix *X*_α_, the nonlinear manifold learning technique: Isomap, was employed. Unlike linear methods, Isomap preserves the intrinsic geometry of the EEG data by maintaining geodesic distances between points on the underlying manifold. This algorithm consists of three major steps (Tenenbaum et al., 2000):

#### 3.2.1 Construction of neighborhood graph

To preserve the local geometry of the EEG data, the *k*-nearest neighbors (*k*-NN) approach is employed. In this step, a graph is constructed by connecting each segment (time window) to its *k* nearest neighbors using the Euclidean distance. The pair wise Euclidean distance is calculated using *X*_α_. The pairwise Euclidean distance between time windows *i* and *j* is:


$$\begin{array}{c} D_{ij}=||X_{i}-X_{j}||,\;i,j=1,2,\ldots ,n. \end{array}$$ (2)

where *X*_*i*_ and *X*_*j*_ in Equation 2 denotes the vectors corresponding to time windows *i* and *j*, respectively. The distance matrix *D*∈ℝ^*n*×*n*^ contains all pairwise distances. For each point *i*, its *k*-nearest neighbors *N*_*k*_(*i*) are determined by selecting the *k* smallest entries in the distance matrix *D*. Using this, an adjacency matrix *W* is constructed using Equation 3.


$$W_{ij}=\{\begin{array}{ll} D_{ij}, & \text{if }j\in N_{k}(i)\text{ or }i\in N_{k}(j),\\ \infty , & \text{otherwise}. \end{array}$$ (3)

The resulting adjacency matrix *W*∈ℝ^*n*×*n*^ encodes neighborhood relations among the *n* time windows: *W*_*ij*_ < ∞ when window *j* is among the *k*-nearest neighbors of *i* (or vice versa), and *W*_*ij*_ = ∞ otherwise. The connectivity of the resulting graph is then verified, and the optimal value of *k* is selected such that the graph becomes fully connected. The procedure for determining this optimal *k* is outlined in Algorithm 1.

Algorithm 1 Optimal *k* value selection. 
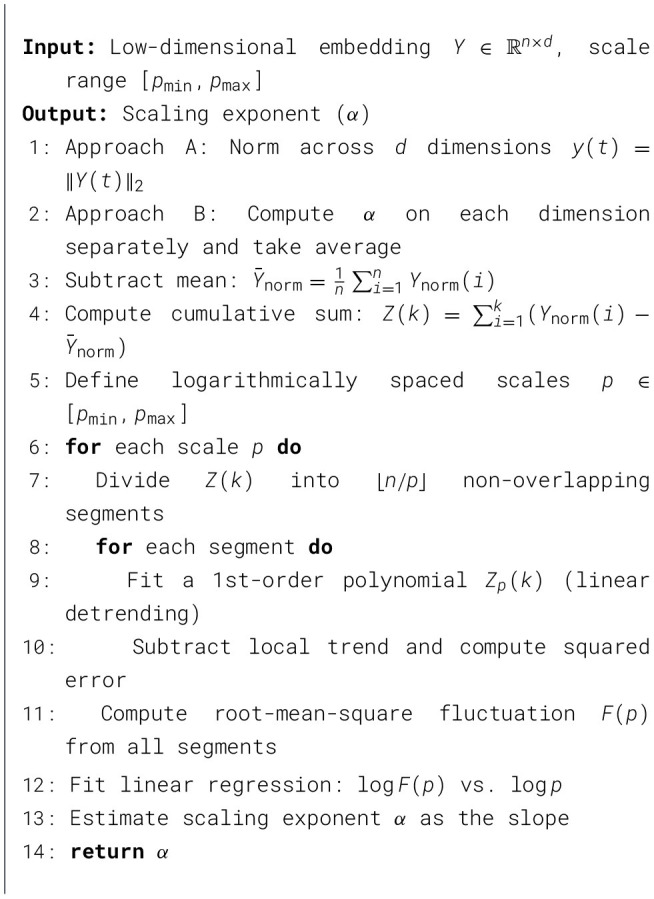

In Algorithm 1, the initial value is chosen as $k_{0}=\sqrt{n}$ as a heuristic initialization (Zhang, 2016). The symbols *V* and *E* denote the set of vertices (time windows) and edges (connections) of the graph, respectively. The neighborhood size *k* is selected adaptively rather than being fixed. Starting from *k*_0_, the value of *k* was incrementally increased until the k-nearest neighbor graph becomes fully connected. Among all such values, the smallest *k* ensuring connectivity is selected.

#### 3.2.2 Estimation of geodesic distances

The goal of this subsection is to find the true geodesic distances by computing the shortest paths on the neighborhood graph (*W*). The geodesic distance matrix (*G*) is computed using Dijkstra's algorithm (Dijkstra, 1959, 2022). This matrix *G*∈ℝ^*n*×*n*^ represents the geodesic distances between all pairs of time windows.

#### 3.2.3 Construction of low-dimensional embedding

In this step, the aim is to obtain a low-dimensional representation of the EEG data, *Y*∈ℝ^*n*×*d*^, where *d* is the required lower dimensions while preserving the geodesic distances. Classical multidimensional scaling (MDS) is performed on the geodesic distance matrix (*G*) to achieve this. Compute the kernel matrix (*B*) using the Equation 4.


$$\begin{array}{c} B=-\frac{1}{2}HG^{2}H \end{array}$$ (4)

where $G^{2}={\left(G_{ij}\right)}^{2}$, and *H* is the centering matrix, given by


$$\begin{array}{c} H=I-\frac{1}{n}\text{1}{\text{1}}^{⊤} \end{array}$$ (5)

The identity matrix (*I*) in Equation 5 is of size *n*×*n*, and **11**^⊤^ denotes the matrix of all-ones, and of size *n*×*n*. By performing the eigen decomposition of the kernel matrix *B*, we obtain


$$\begin{array}{c} B=V\Lambda V^{⊤} \end{array}$$ (6)

where Λ and *V* in Equation 6 denote the eigenvalues and eigenvectors of *B*, respectively. Select the top *d* eigenvalues and eigenvectors, and form the embedding matrix *Y* as,


$$\begin{array}{c} Y=V_{d}\Lambda_{d}^{1/2} \end{array}$$ (7)

From Equation 7, the original EEG data $X_{\alpha }\in {\mathbb{R}}^{n\times N}$ is reduced to a lower-dimensional embedding matrix *Y*∈ℝ^*n*×*d*^. The complete algorithm of Isomap is presented in Algorithm 2.

Algorithm 2 Isomap dimensionality reduction. 
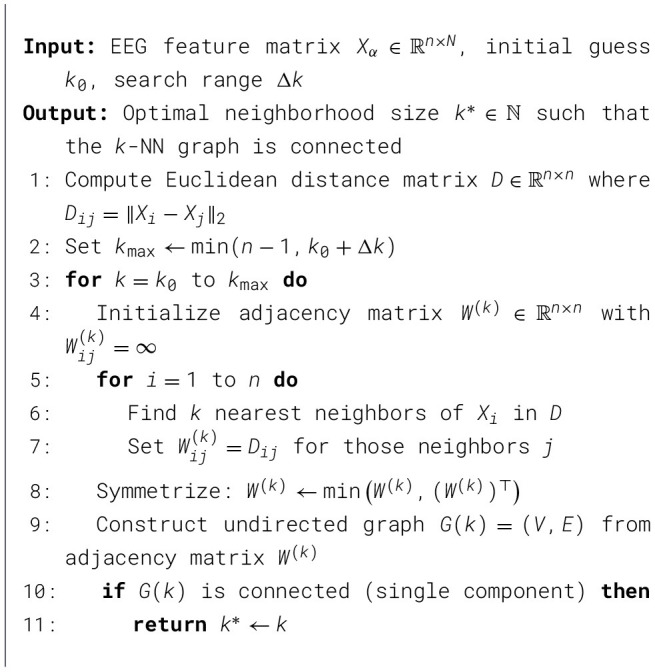

### 3.3 Application of DFA algorithm

From Equation 7, the low-dimensional embedding matrix *Y*∈ℝ^*n*×*d*^ represents the global structure of the EEG data in a reduced *d*-dimensional space. Two approaches can be considered for the DFA analysis: (i) applying DFA to a one-dimensional signal obtained by taking the Euclidean norm across the *d* dimensions, (ii) applying DFA separately to each dimension of *Y* and averaging the resulting scaling exponents (α).

The mathematical formulation below is presented for the norm-based approach, while the detailed formulation of the mean-based approach is provided in the Appendix.

For applying the proposed DFA algorithm, the low-dimensional embedding matrix *Y*∈ℝ^*n*×*d*^ was converted into a one-dimensional representation of size *n*×1 by computing the Euclidean norm across the *d* dimensions.


$$\begin{array}{c} Y_{\text{norm}}\left(i\right)=\sqrt{\overset{d}{\underset{j=1}{\sum }}Y_{ij}^{2}},\;i=1,2,\ldots ,n, \end{array}$$ (8)

*Y*∈ℝ^*n*×*d*^ in Equation 8 is the low-dimensional embedding, and $Y_{\text{norm}}\in {\mathbb{R}}^{n\times 1}$ is the resulting one-dimensional time series. This is then used as the input for DFA.

The DFA technique has three major steps (Peng et al., 1994). The first step is, mean removal and cumulative sum. For the given signal *Y*_norm_(*i*), *i* = 1, 2, …, *n*, the mean is defined as


$$\begin{array}{c} {Ȳ}_{\text{norm}}=\frac{1}{n}\overset{n}{\underset{i=1}{\sum }}Y_{\text{norm}}\left(i\right). \end{array}$$ (9)

The cumulative sum after removal of the mean is computed using Equation 9 as,


$$Z(k)=\sum_{i=1}^{k}(Y_{\text{norm}}(i)-{\bar{Y}}_{\text{norm}}),\;k=1,2,\ldots ,n.$$ (10)

The second step of the DFA algorithm is the local detrending, in which, the integrated series from Equation 10 *Z*(*k*) is divided into *p* non-overlapping segments of equal length. It is varied within the range of,


$$\begin{array}{c} p\in \left[p_{\min},p_{\max}\right],\;p_{\min}=4,p_{\max}=\left\lfloor \frac{n}{4}\right\rfloor . \end{array}$$ (11)

Within each segment, a least squares polynomial fit *Z*_*p*_(*k*) is computed to represent the local trend. The root-mean-square fluctuation at scale *p* of the integrated series is given by,


$$F(p)=\sqrt{\frac{1}{n}\sum_{k=1}^{n}(Z(k)-Z_{p}(k){)}^{2}}.$$ (12)

*Z*(*k*)−*Z*_*p*_(*k*) in Equation 12 is known as detrending. The range in Equation 11, [4, *n*/4] (Peng et al., 1994) ensures that the analysis includes multiple scales while maintaining statistical reliability, as both very small and very large box sizes are avoided.

The final step of DFA is the scaling behavior. Now, the relationship between the detrended series and the segment lengths given as,


$$\begin{array}{c} F\left(p\right)\propto p^{\alpha } \end{array}$$ (13)

**Figure 2:**
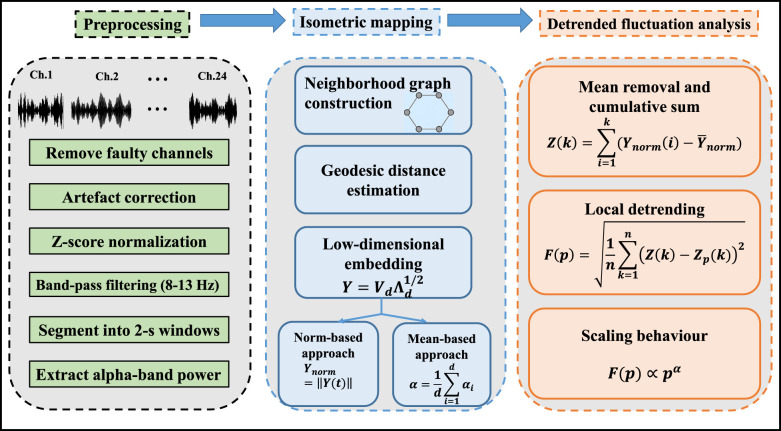
Graphical abstract of the proposed framework showing the preprocessing, Isomap embedding, and DFA analysis steps.

The α in the Equation 13 is known as the scaling exponent and is defined as the slope of the double logarithmic plot log *F*(*p*) vs. log(*p*). The complete DFA on the low-dimensional embedding is presented in Algorithm 3. The scaling exponent (α) provides the long-range temporal correlation (LRTC) of the EEG data. When α = 0.5, it indicates the EEG signal is completely uncorrelated and represents white noise. When long-range temporal correlations are analyzed in EEG signals, a power law pattern typically produces scaling exponents between 0.5 and 1. Values closer to 1 indicate stronger persistence, indicating the correlations decay more slowly over time. If the exponent exceeds 1, the relationship no longer follows a power law, and LRTC is not preserved (Linkenkaer-Hansen et al., 2001). The complete bird's-eye view of the proposed methodology is shown in Figure 2.

Algorithm 3 Detrended fluctuation analysis (DFA). 
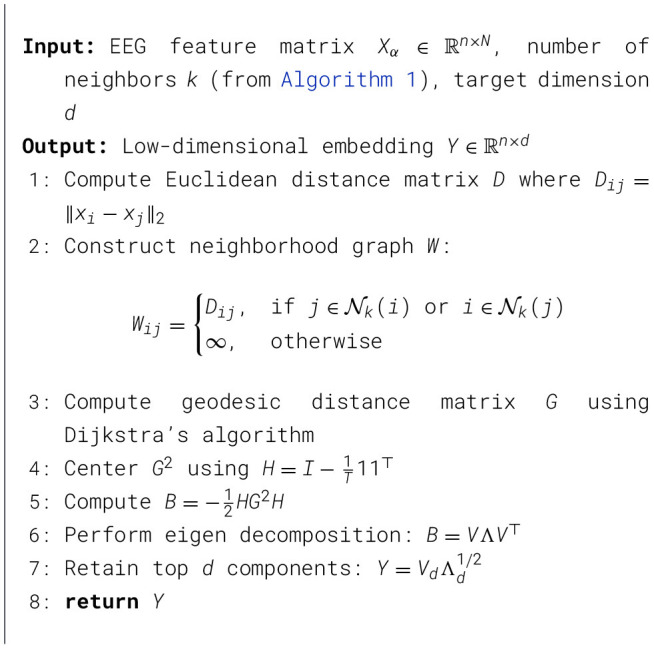

## 4 Results

### 4.1 Selection of lower-dimensional embedding

Finding the appropriate number of lower dimensions after constructing the embedding matrix (Equation 7) is crucial for the proposed DFA analysis. To determine the optimal embedding dimension, the elbow method was employed on the eigenvalue spectrum of the kernel matrix *B* (Equation 6). This method identifies the point where the eigenvalue decay curve exhibits a clear inflection, suggesting, the additional dimensions contribute only marginally to the variance.

**Figure 3:**
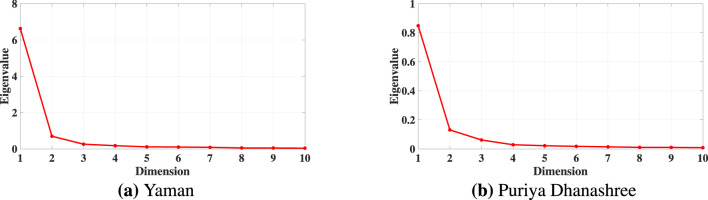
Eigenvalue spectra of the kernel matrix (*B*) for Subject 12. The elbow after the third eigenvalue indicates an optimal embedding dimension of *d* = 3. **(a)** Yaman. **(b)** Puriya Dhanashree.

Figure 3 illustrates the eigenvalue spectra for both ragas in subject 12. In both ragas, the curve exhibits a clear elbow after the third dimension, beyond which the eigenvalues level off. Based on this observation, we have selected three (3) dimensions for the reduced embedding. Figures 3a, b demonstrate the eigenvalue spectrum behavior for both the ragas (Yaman and Puriya Dhanashree, respectively). The similarity in the eigenvalue spectral decay in both ragas supports the robustness of choosing a three-dimensional embedding. This ensures the essential geometric structure of the EEG data is preserved while minimizing redundancy and noise. While Figure 3 shows the eigenvalue spectrum for one subject, consistent spectral patterns were observed in all subjects and both ragas (refer to Supplementary File 1). This consistency supports the adoption of *d* = 3 as the embedding dimension throughout.

To provide a more quantitative comparison, we additionally evaluated neighborhood preservation using the trustworthiness metric (Venna and Kaski, 2001) and variance explained (Tables 2, 3). From Table 2, it is observed that PCA shows higher trustworthiness values (≈0.98–0.99), which reflects the preservation of local Euclidean structure. In contrast, Isomap achieves consistently high trustworthiness (≈0.94–0.96), indicating robust neighborhood preservation while additionally capturing nonlinear geometric structure. The trustworthiness metric was computed using neighborhood sizes *K*_*t*_ = 5 and *K*_*t*_ = 7, where *K*_*t*_ denotes the number of nearest neighbors considered for the local structure preservation. These values were selected based on prior studies (Venna and Kaski, 2001; Van Der Maaten et al., 2009), where small neighborhood sizes (5–15) are commonly used to assess local neighborhood preservation. Furthermore, the three-dimensional (*d* = 3) embedding captures a substantial portion of the variance, with mean explained variance of 80.02%±8.12% for Raga Yaman and 82.30%±8.96% for Raga Puriya Dhanashree. For nonlinear methods such as Isomap, variance explained alone is not sufficient to characterize embedding quality, as the method is designed to preserve intrinsic geometric structure rather than maximize variance.

**Table 2:** Trustworthiness (Mean ± Std) for both the methods PCA and Isomap during and after music listening for Raga Yaman and Raga Puriya Dhanashree.

Method	*K*_*t*_	Yaman		Puriya Dhanashree	
		Durin	After	During	After
Isomap	5	0.957 ± 0.015	0.937 ± 0.034	0.956 ± 0.014	0.935 ± 0.032
	7	0.961 ± 0.014	0.944 ± 0.030	0.960 ± 0.014	0.936 ± 0.034
PCA	5	0.993 ± 0.005	0.984 ± 0.016	0.993 ± 0.004	0.984 ± 0.012
	7	0.994 ± 0.004	0.986 ± 0.014	0.994 ± 0.005	0.986 ± 0.010

**Table 3:** Cumulative explained variance (%) captured by the first three dimensions (*d* = 3) for all subjects across Raga Yaman and Raga Puriya Dhanashree.

Subject	Yaman (%)	Puriya Dhanashree (%)
1	80.15	79.40
2	84.86	97.47
3	82.74	98.84
4	77.02	76.70
5	74.55	88.21
6	85.39	86.65
7	89.59	86.79
8	77.33	80.83
9	73.22	74.06
10	60.47	72.11
11	86.84	75.01
12	89.62	85.29
13	78.54	72.54

### 4.2 Temporal scaling exponent (α)

The temporal scaling exponent (α) characterizes the strength of long-range temporal correlations (LRTC) in the EEG dynamics.

**Figure 4:**
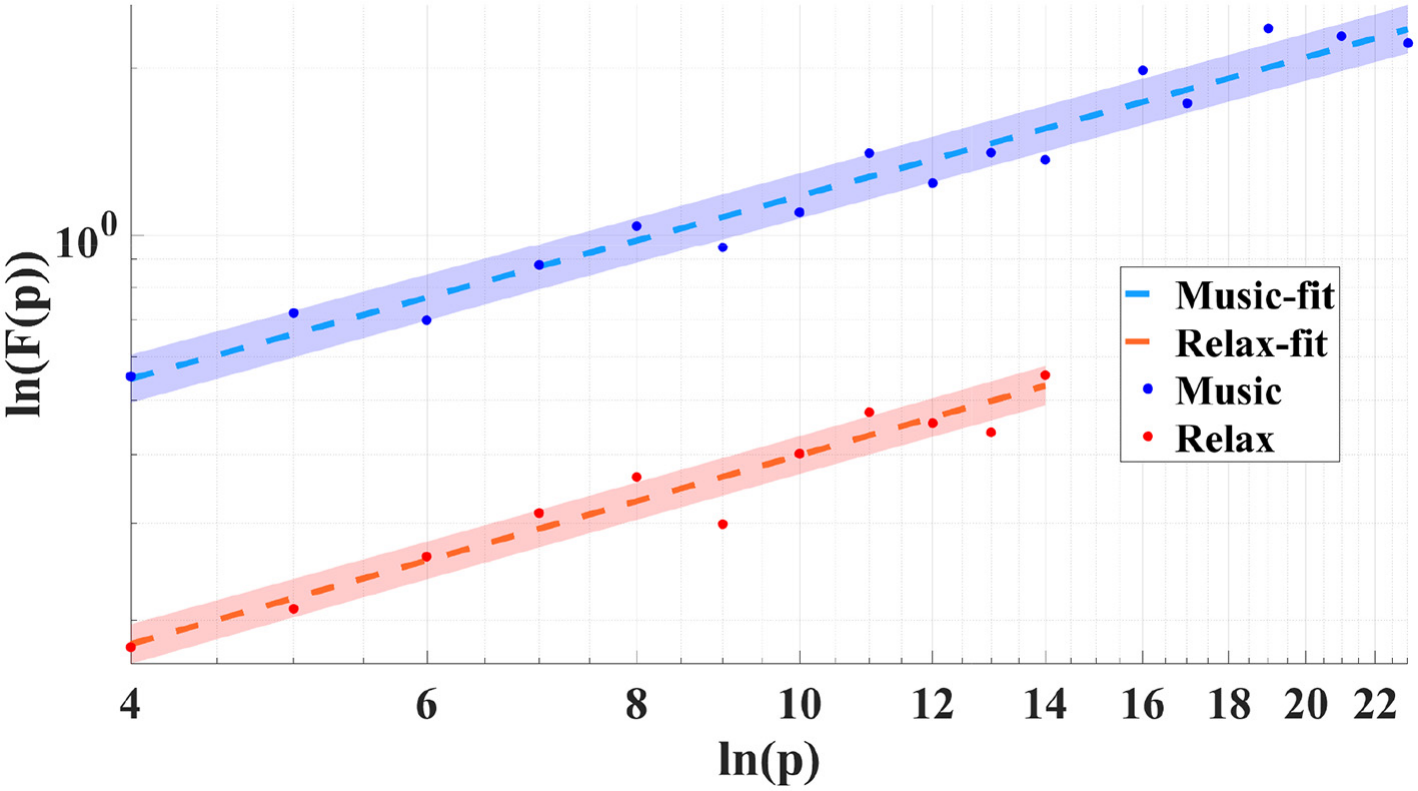
Log–log plot of the fluctuation function *F*(*p*) vs. scale *p* for raga Yaman showing the linear fit for both the conditions: during the music and relaxation (Subject 13).

**Figure 5:**
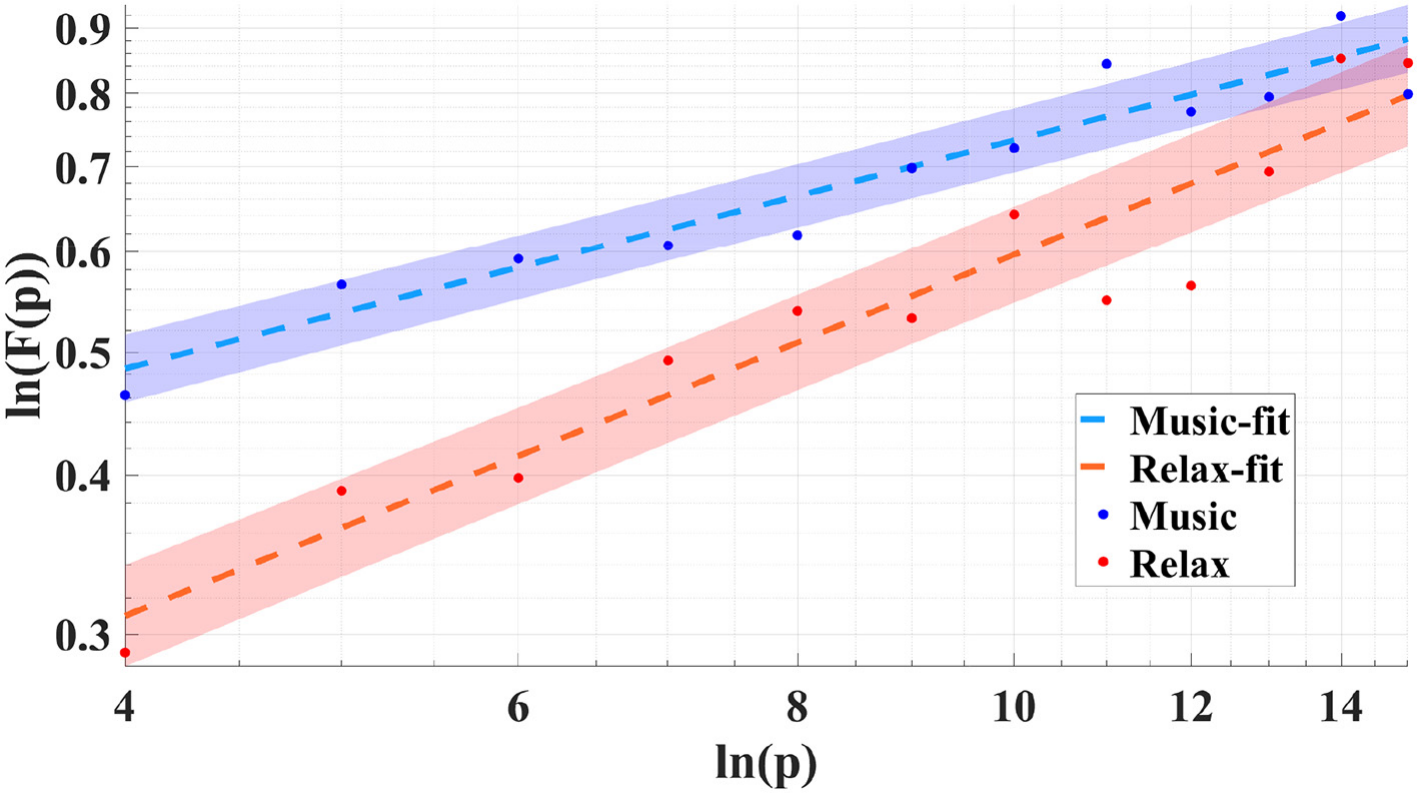
Log–log plot of the fluctuation function *F*(*p*) vs. scale *p* for raga Puriya Dhanashree showing the linear fit for both the conditions: during the music and relaxation (Subject 10).

The deviations of α from the white noise baseline quantify the degree to which the EEG exhibits temporal self-similarity. Figures 4, 5 illustrate the scaling behavior by showing the log–log dependence of the fluctuation function *F*(*p*) on the scale *p* for both the ragas Yaman and Puriya Dhanashree, respectively. For each raga, the results are presented for both the music and relaxation conditions. The linear trends demonstrate the presence of scale-free dynamics, while the slopes of the fitted lines yield the scaling exponents (α). The differences between the music and relaxation conditions are reflected in distinct α values, indicating that musical stimulation modulates the persistence of temporal correlations in EEG activity.

The scaling exponent (α) exhibited inter-subject variability, reflecting individual differences in responses to music. Table 4 reports the α values across all subjects for both the ragas and conditions (music, relaxation) for both approaches. The distribution of values in both approaches demonstrates that individual subjects have distinct temporal scaling behaviors under each condition. Nevertheless, for most subjects, the α is within the interval 0.5 < α < 1, indicative of persistent long-range temporal correlations in the EEG dynamics.

**Table 4:** Scaling exponent (α) during and after music listening for both the ragas Yaman and Puriya Dhanashree using both the approaches (Isomap).

Subjects	Norm-based approach				Mean-based approach			
	Yaman		Puriya Dhanashree		Yaman		Puriya Dhanashree	
	During	After	During	After	During	After	During	After
1	0.48	0.40	0.59	1.66	0.74	0.62	0.60	0.89
2	0.55	0.65	0.71	0.53	0.52	0.79	0.71	0.60
3	0.60	0.49	1.31	0.48	0.62	0.50	0.75	0.53
4	0.78	0.99	0.74	0.62	0.78	0.84	0.54	0.56
5	0.54	0.89	0.53	0.62	0.57	0.60	0.62	0.57
6	0.95	0.83	0.39	1.00	0.62	0.66	0.58	0.76
7	0.76	0.70	0.53	0.52	0.79	0.74	0.80	0.62
8	0.44	0.65	0.54	0.86	0.51	0.95	0.57	0.88
9	0.42	0.80	0.79	0.60	0.48	0.63	0.75	0.64
10	0.72	0.43	0.45	0.71	0.67	0.74	0.84	0.62
11	0.77	0.66	0.59	0.95	0.80	0.63	0.66	0.67
12	0.71	0.63	1.28	0.64	0.73	0.56	0.90	0.77
13	0.83	0.85	0.62	1.42	0.72	0.83	0.57	0.86

### 4.3 Comparison with linear dimensionality reduction technique

To assess the robustness of the proposed nonlinear embedding, we performed an equivalent analysis using the linear dimensionality reduction technique: principal component analysis (PCA). The same preprocessing and alpha-band (8–13 Hz) power extraction pipeline was employed as in the Isomap analysis. PCA was then performed on the feature matrix $X_{\alpha }\in {\mathbb{R}}^{n\times N}$ to obtain a linear lower-dimensional embedding. The effective dimensionality was determined using the elbow method on eigenvalue spectrum, which identifies the number of principal components required to capture the maximum variance.

**Figure 6:**
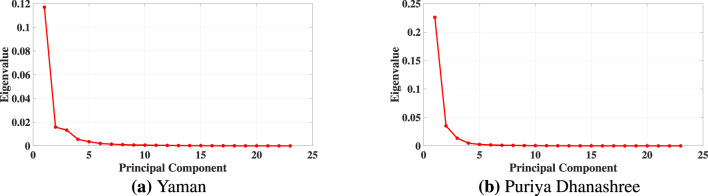
Eigenvalue spectra after performing PCA for Subject 5. The elbow after the fifth eigenvalue indicates an optimal embedding dimension of *d* = 5. **(a)** Yaman. **(b)** Puriya Dhanashree.

Figure 6 shows the PCA eigenvalue spectra, with Figures 6a, b corresponding to the ragas Yaman and Puriya Dhanashree, respectively. In both the ragas, the eigenvalue spectrum decays with a clear elbow after the fifth (5) component. Hence, a five-dimensional linear embedding was selected uniformly across all subjects (Supplementary File 2).

After fixing the linear embedding dimension as five, we applied the same procedure as for the Isomap: (i) Norm-based approach and (ii) Mean-based approach. The scaling exponents α obtained across all subjects for both ragas and conditions from both approaches are reported in Table 5. It shows that for most subjects, the α lies within the interval 0.5 < α < 1, consistent with persistent long-range temporal correlations (LRTC).

**Table 5:** Scaling exponent (α) during and after music listening for both the ragas Yaman and Puriya Dhanashree using PCA-DFA.

Subjects	Norm-based approach				Mean-based approach			
	Yaman		Puriya Dhanashree		Yaman		Puriya Dhanashree	
	During	After	During	After	During	After	During	After
1	0.48	0.41	0.55	1.62	0.65	0.60	0.64	0.67
2	0.56	0.67	0.73	0.54	0.52	0.70	0.57	0.57
3	0.59	0.50	1.33	0.49	0.59	0.61	0.70	0.66
4	0.74	1.04	0.70	0.62	0.71	0.68	0.54	0.61
5	0.53	0.92	0.55	0.57	0.59	0.62	0.59	0.57
6	0.97	0.84	0.41	0.96	0.55	0.58	0.56	0.71
7	0.80	0.73	0.62	0.48	0.70	0.72	0.68	0.64
8	0.44	0.67	0.57	0.83	0.53	0.71	0.59	0.77
9	0.43	0.84	0.80	0.59	0.52	0.70	0.67	0.61
10	0.62	0.49	0.49	0.70	0.68	0.67	0.70	0.63
11	0.75	0.64	0.53	0.95	0.75	0.66	0.61	0.66
12	0.69	0.55	1.32	0.65	0.69	0.58	0.74	0.73
13	0.81	0.87	0.60	1.43	0.61	0.77	0.56	0.80

## 5 Discussion

Tables 4, 5 show that, for both embeddings (Isomap and PCA), a few subjects exhibited α values greater than 1 and less than 0.5 in the norm-based approach, indicating a deviation from long-range temporal correlation (LRTC) behavior. This behavior may arise from the construction of the one-dimensional signal using the Euclidean norm across embedding dimensions. Specifically, the norm operation aggregates all dimensions into a single magnitude-based signal, which can amplify fluctuations and introduce distortions. As a result, the temporal structure of the signal may be altered, leading to instability in the Detrended Fluctuation Analysis (DFA) scaling behavior and deviations from the expected LRTC regime. This highlights a limitation of the norm-based approach for capturing temporal dynamics. However, such values were rarely observed in the mean-based approach for either embedding method, suggesting that the mean-based approach provides more stable and reliable estimates of α in both linear and nonlinear embeddings.

The mean values of the scaling exponent (α) and the corresponding goodness of fit (*R*^2^) obtained from the nonlinear Isomap embedding are summarized in Table 6 for both the approaches. For both ragas and conditions, the average scaling exponent (α) is between 0.66 and 0.82 for the norm-based approach, between 0.66 and 0.70 for the mean-based approach. The two approaches are within the range of persistent long-range temporal correlations (0.5 < α < 1). This indicates that the EEG dynamics retain temporal correlations across multiple time scales, a hallmark of scale-free organization in complex systems. Raga Puriya Dhanashree yielded higher mean α values than raga Yaman for both approaches, except during relaxation in the mean-based approach. This suggests the intrinsic temporal correlations are stronger for this musical stimulus.

**Table 6:** Scaling exponent (α) and goodness of fit (*R*^2^) values (Mean ± Std) during music and relaxation conditions for both ragas using norm-based and mean-based approaches (Isomap).

Raga	Condition	Alpha (Mean ± Std)		*R*^2^ (Mean ± Std)	
		Norm-based	Mean-based	Norm-based	Mean-based
Yaman	During music	0.66 ± 0.16	0.66 ± 0.11	0.91 ± 0.05	0.93 ± 0.02
	Relaxation	0.69 ± 0.18	0.70 ± 0.12	0.72 ± 0.21	0.83 ± 0.07
Puriya Dhanashree	During music	0.70 ± 0.28	0.68 ± 0.11	0.90 ± 0.05	0.90 ± 0.04
	Relaxation	0.82 ± 0.36	0.69 ± 0.12	0.87 ± 0.07	0.84 ± 0.08

Furthermore, for both ragas, the relaxation condition showed larger mean α values compared to the music conditions. This persistence after stimulus removal implies a prolonged temporal memory in the underlying neural dynamics, consistent with the idea that stimulus-induced states leave a trace in the brain. The consistently higher *R*^2^ values from both approaches across all conditions and ragas indicate that the fluctuation functions follow a robust power law scaling, reinforcing the validity of the fractal characterization in the embedded space. The norm-based approach yielded *R*^2^ values in the range of 0.72–0.91, while the mean-based approach showed a slightly higher range of 0.83–0.93. These findings support the view that EEG dynamics under musical stimulus exhibit nonlinear structure and persistent long-range correlations.

The mean scaling exponent (α) and the corresponding goodness of fit values (*R*^2^) obtained from the linear embedding (PCA) are summarized in Table 7. For the norm-based approach, the mean α values (0.65 < α < 0.80) lie within the LRTC range (0.5 < α < 1) for both ragas and the conditions. For the mean-based approach, the alpha values are in the range (0.62 < α < 0.66). Similar to Isomap, PCA embedding showed greater alpha values during the relaxation condition for both ragas and approaches. It also showed that raga Puriya Dhanashree consistently yielded higher mean α values compared to raga Yaman.

**Table 7:** Scaling exponent (α) and goodness of fit (*R*^2^) values (Mean ± Std) during music and relaxation conditions for both ragas using norm-based and mean-based approaches (PCA).

Raga	Condition	Alpha (Mean ± Std)		*R*^2^ (Mean ± Std)	
		Norm-based	Mean-based	Norm-based	Mean-based
Yaman	During music	0.65 ± 0.16	0.62 ± 0.07	0.90 ± 0.06	0.93 ± 0.01
	Relaxation	0.71 ± 0.19	0.66 ± 0.05	0.70 ± 0.21	0.82 ± 0.06
Puriya Dhanashree	During music	0.71 ± 0.29	0.63 ± 0.06	0.90 ± 0.06	0.89 ± 0.05
	Relaxation	0.80 ± 0.36	0.66 ± 0.07	0.85 ± 0.08	0.83 ± 0.11

From Tables 6, 7, it is evident that the mean scaling exponent (α) is consistently larger during relaxation than during music condition in both approaches. This increase in α indicates stronger LRTCs, in line with prior studies that established the presence of scale-free dynamics in EEG (Linkenkaer-Hansen et al., 2001; Hardstone et al., 2012; Smit et al., 2013). Furthermore, recent work has demonstrated that LRTC tend to be enhanced in relaxed or post-stimulus states compared to active task conditions, supporting our observation that relaxation yields higher α values (Teixeira Borges et al., 2019; Wairagkar et al., 2021). Another EEG–DFA study (Pavlov et al., 2018) also reported persistent temporal correlations in task-related EEG activity. Together, these results reinforce the interpretation that the brain retains persistent temporal correlations even after the external musical stimulus is removed. The relatively higher variability in *R*^2^ observed for the relaxation condition in raga Yaman (norm-based approach) is consistent with the wider spread of α values across subjects, indicating less consistent scaling behavior and increased inter-subject variability in this condition. To ensure a fair comparison between the linear and nonlinear techniques, we computed scaling exponents (α) using Isomap with *d* = 5, matching the PCA dimensionality. The mean values, along with standard deviations, for both approaches are presented in Table 9 of the Supplementary File 3. The results show that α values from Isomap (*d* = 5) are nearly identical to those from PCA (*d* = 5), with differences less than 0.02 and overlapping standard deviations across all conditions, indicating that both methods yield comparable scaling exponent estimates at matched dimensionality. It is also observed that the mean-based scaling exponent (α) values obtained using Isomap with *d* = 3 were slightly higher than those obtained with *d* = 5, indicating a sensitivity of this measure to the choice of embedding dimensionality.

A key difference between our findings and Banerjee et al. (2016) lies in the interpretation of the DFA results. The authors of Banerjee et al. (2016) reported that the increase in fractal dimension (FD) during music reflected greater neural “complexity” or arousal. However, since FD = 3-α, their higher FD values actually correspond to lower DFA exponents (α). When converted, their own data show that α decreased to 0.7 to 0.9 during the music condition: precisely the range that indicates persistent LRTC. The baseline and post-music conditions yielded α>1, outside the regime of LRTC. Thus, their interpretation that music increases “complexity” is not fully aligned with the standard DFA theory. Our approach resolves this issue by analyzing α values directly, within a multichannel embedding space that accounts for spatial correlations across EEG channels. This yields stable α values in the physiologically meaningful 0.5 to 1 range (LRTC). Thereby providing a more robust and theoretically consistent characterization of scale-free brain dynamics during music listening.

From Figures 3, 6, it is evident that the nonlinear Isomap embedding required only three dimensions to capture the data's underlying structure, whereas the linear PCA embedding required five. This difference arises because EEG dynamics evolve on a nonlinear manifold, and Isomap, by relying on geodesic distances, is able to preserve the intrinsic geometry of the system effectively. Although the scaling exponents (α) obtained from both linear (PCA) and nonlinear (Isomap) embeddings show similar trends, the Isomap representation achieves the same with fewer dimensions and yields slightly improved values of the scaling exponent (α) as well as higher goodness of fit (*R*^2^) compared to PCA in the mean-based approach.

Consistent with the above findings, the mean α values were higher during relaxation than during the music condition, suggesting stronger long-range temporal correlations in the relaxation state. However, these differences did not reach statistical significance, likely reflecting the limited sample size. To compare analytical approaches, we employed paired-samples *t*-tests. Among the different approaches, only the Isomap mean-based DFA yielded statistically significant (α) value differences compared to the PCA mean-based DFA approach during music listening for both ragas (Yaman: *p* = 0.02; Puriya Dhanashree: *p* = 0.008). Bonferroni correction was applied to account for multiple comparisons, after which the difference remained statistically significant for Puriya Dhanashree (*p* = 0.035), while the result for Yaman did not reach statistical significance (*p* = 0.096). The effect size analysis indicated a medium-to-large effect for Yaman (Cohen's *d* = 0.72) and a large effect for Puriya Dhanashree (Cohen's *d* = 0.87), demonstrating that the observed differences are not only statistically significant but also of moderate to large magnitude. We also used the Wilcoxon signed-rank test as a non-parametric alternative to the paired *t*-test for comparing the scaling exponent α values. The Wilcoxon signed-rank test showed statistically significant differences for both ragas (Yaman: *p* = 0.027; Puriya Dhanashree: *p* = 0.010), which are consistent with the paired *t*-test results. No significant differences were observed during relaxation. The lack of statistical significance in the relaxation conditions does not necessarily indicate the absence of an effect. Instead, it may reflect limited statistical power given the relatively small sample size. These results suggest that combining nonlinear manifold embeddings with mean-based DFA enhances the ability to detect music-related changes in temporal correlations. Further studies with larger sample sizes are needed to determine whether the relaxation trends consistently achieve statistical significance.

To evaluate the robustness of the estimated scaling exponents (α), we conducted a moving-block bootstrap analysis with 1000 resamples. In this procedure, contiguous blocks of the windowed EEG feature time series were resampled with replacement, with block length selected using a heuristic proportional to the square root of the number of windows (rounded to the nearest integer, with a minimum of 2) (Lahiri, 2013). This choice provides a practical balance between preserving temporal dependence and maintaining sufficient variability across resampled blocks. More rigorous, data-driven approaches for selecting an optimal block length could be explored in future work.

This nonparametric approach provides an estimate of variability and allows the construction of confidence intervals, thereby assessing the stability of the observed effects across subjects and conditions. The mean α values and corresponding 95% confidence intervals (CIs) for the mean-based and norm-based approaches under both Isomap and PCA embeddings are reported in Tables 8, 9. Comparison with the original DFA estimates (Tables 4, 5) shows that the bootstrap means closely match the original α values across subjects. Moreover, in most cases, the original α estimates lie within the 95% CIs, confirming the consistency of the results. Notably, for the norm-based approach (both PCA and Isomap), the lower bound of the CI was negative in a subset of subjects, whereas this was not observed for the mean-based approach. The presence of negative confidence intervals suggests instability in the estimation process, particularly in the norm-based approach. This is consistent with the broader observation in our study that the norm-based approach exhibits higher variability compared to the mean-based approach. This finding indicates that the mean-based approach yields more stable and reliable estimates of α compared to the norm-based approach. We note that the moving-block bootstrap employed here preserves temporal dependence but does not explicitly account for cross-scale correlations inherent in DFA, and the block length was selected using a heuristic rather than a data-driven optimality criterion. Therefore, the resulting confidence intervals should be interpreted with caution and may underestimate uncertainty. Such concerns about stability of scaling exponents are consistent with prior complexity research, where Pavlov et al. (2020) demonstrated that noise can significantly affect the scaling exponents.

**Table 8:** Bootstrap estimates of mean scaling exponent (α) with 95% confidence intervals (CIs) for both ragas using both the approaches: norm-based and mean-based (Isomap).

Subject	Norm-based approach				Mean-based approach			
	Yaman		Puriya Dhanashree		Yaman		Puriya Dhanashree	
	During	After	During	After	During	After	During	After
1	0.55 [0.37, 0.74]	0.49 [0.23, 0.84]	0.53 [0.36, 0.74]	0.79 [0.12, 1.59]	0.68 [0.57, 0.79]	0.62 [0.45, 0.82]	0.61 [0.48, 0.73]	0.67 [0.42, 0.90]
2	0.53 [0.33, 0.76]	0.49 [0.18, 0.87]	0.77 [0.33, 1.25]	0.65 [0.36, 0.99]	0.53 [0.43, 0.66]	0.68 [0.50, 0.90]	0.70 [0.39, 0.96]	0.69 [0.47, 0.91]
3	0.58 [0.40, 0.79]	0.62 [0.30, 1.04]	0.91 [0.26, 1.54]	0.50 [0.27, 0.79]	0.61 [0.46, 0.77]	0.55 [0.40, 0.68]	0.61 [0.39, 0.81]	0.61 [0.44, 0.78]
4	0.74 [0.46, 1.00]	0.81 [0.36, 1.26]	0.58 [0.34, 0.86]	0.65 [0.10, 1.46]	0.73 [0.57, 0.89]	0.71 [0.48, 0.90]	0.56 [0.45, 0.68]	0.60 [0.39, 0.86]
5	0.59 [0.38, 0.82]	0.58 [0.39, 0.83]	0.55 [0.28, 0.92]	0.75 [0.43, 1.13]	0.57 [0.45, 0.71]	0.51 [0.35, 0.67]	0.62 [0.47, 0.78]	0.61 [0.46, 0.79]
6	0.70 [0.35, 1.00]	0.70 [0.28, 1.20]	0.50 [0.29, 0.76]	0.85 [−0.72, 2.77]	0.5676 [0.42, 0.68]	0.64 [0.42, 0.86]	0.5918 [0.44, 0.75]	0.78 [0.30, 1.31]
7	0.80 [0.48, 1.08]	0.59 [0.36, 0.85]	0.52 [0.31, 0.77]	0.68 [0.46, 1.00]	0.78 [0.59, 0.95]	0.76 [0.57, 0.99]	0.65 [0.51, 0.83]	0.68 [0.48, 0.87]
8	0.47 [0.33, 0.63]	0.55 [0.28, 0.94]	0.51 [0.35, 0.71]	0.66 [0.37, 0.98]	0.52 [0.44, 0.61]	0.81 [0.59, 1.06]	0.54 [0.45, 0.65]	0.75 [0.56, 0.95]
9	0.45 [0.29, 0.62]	0.59 [−0.08, 1.22]	0.63 [0.40, 0.85]	0.65 [0.42, 0.91]	0.49 [0.38, 0.60]	0.61 [0.45, 0.81]	0.60 [0.50, 0.70]	0.60 [0.46, 0.75]
10	0.67 [0.50, 0.84]	0.57 [0.35, 0.82]	0.59 [0.26, 0.97]	0.66 [0.39, 0.92]	0.62 [0.51, 0.72]	0.67 [0.52, 0.85]	0.82 [0.63, 1.03]	0.65 [0.48, 0.84]
11	0.66 [0.44, 0.90]	0.70 [0.36, 1.27]	0.63 [0.43, 0.88]	0.62 [−0.35, 1.44]	0.68 [0.56, 0.80]	0.70 [0.48, 0.96]	0.64 [0.51, 0.79]	0.64 [0.30, 0.93]
12	0.65 [0.44, 0.89]	0.68 [0.43, 0.94]	0.90 [0.39, 1.37]	0.63 [0.34, 0.96]	0.68 [0.54, 0.84]	0.58 [0.45, 0.70]	0.73 [0.48, 0.94]	0.73 [0.54, 0.91]
13	0.79 [0.60, 0.97]	0.64 [0.28, 0.96]	0.59 [0.35, 0.88]	0.88 [0.42, 1.40]	0.70 [0.57, 0.83]	0.74 [0.54, 0.92]	0.61 [0.52, 0.71]	0.73 [0.48, 0.99]

For each subject mean α is shown on the first line and the 95% CI on the second line.

**Table 9:** Mean scaling exponent (α) with 95% confidence intervals (CIs) obtained using bootstrap for both ragas from both the approaches: norm-based and mean-based (PCA).

Subject	Norm-based approach				Mean-based approach			
	Yaman		Puriya Dhanashree		Yaman		Puriya Dhanashree	
	During	After	During	After	During	After	During	After
1	0.55 [0.38,0.03]	0.51 [0.24,0.86]	0.52 [0.34,0.73]	0.74 [–0.004,1.57]	0.63 [0.54,0.71]	0.65 [0.50,0.79]	0.62 [0.51,0.73]	0.62 [0.39,0.81]
2	0.53 [0.34,0.77]	0.49 [0.18,0.89]	0.78 [0.31,1.29]	0.66 [0.36,1.01]	0.54 [0.46,0.63]	0.66 [0.52,0.82]	0.64 [0.41,0.81]	0.62 [0.44,0.79]
3	0.57 [0.38,0.77]	0.64 [0.32,1.05]	0.92 [0.23,1.56]	0.51 [0.28,0.81]	0.57 [0.47,0.67]	0.58 [0.45,0.71]	0.65 [0.47,0.82]	0.64 [0.48,0.81]
4	0.70 [0.42,0.97]	0.82 [0.35,1.30]	0.54 [0.29,0.83]	0.69 [0.11,1.59]	0.66 [0.54,0.76]	0.69 [0.50,0.85]	0.57 [0.48,0.67]	0.62 [0.46,0.81]
5	0.58 [0.36,0.84]	0.59 [0.38,0.85]	0.57 [0.27,1.00]	0.71 [0.39,1.12]	0.59 [0.49,0.70]	0.53 [0.41,0.66]	0.59 [0.48,0.72]	0.61 [0.48,0.74]
6	0.70 [0.36,0.99]	0.68 [0.19,1.35]	0.50 [0.29,0.76]	0.87 [–0.89,2.91]	0.54 [0.45,0.61]	0.59 [0.39,0.79]	0.59 [0.48,0.72]	0.70 [0.32,1.17]
7	0.82 [0.47,1.11]	0.61 [0.36,0.86]	0.60 [0.38,0.84]	0.67 [0.43,1.01]	0.67 [0.56,0.78]	0.72 [0.59,0.85]	0.60 [0.50,0.71]	0.66 [0.53,0.79]
8	0.45 [0.31,0.62]	0.54 [0.28,0.93]	0.53 [0.37,0.71]	0.62 [0.31,0.94]	0.54 [0.46,0.61]	0.70 [0.55,0.86]	0.57 [0.50,0.65]	0.64 [0.50,0.78]
9	0.46 [0.30,0.66]	0.60 [–0.11,1.27]	0.63 [0.36,0.87]	0.65 [0.40,0.91]	0.51 [0.42,0.61]	0.59 [0.46,0.74]	0.57 [0.48,0.66]	0.55 [0.43,0.66]
10	0.60 [0.44,0.75]	0.58 [0.36,0.85]	0.61 [0.29,0.97]	0.63 [0.34,0.90]	0.63 [0.55,0.71]	0.64 [0.52,0.78]	0.67 [0.55,0.80]	0.68 [0.51,0.83]
11	0.65 [0.43,0.89]	0.73 [0.40,1.36]	0.58 [0.41,0.82]	0.61 [–0.49,1.47]	0.64 [0.54,0.73]	0.68 [0.45,0.91]	0.60 [0.49,0.72]	0.63 [0.39,0.82]
12	0.63 [0.43,0.84]	0.64 [0.41,0.91]	0.91 [0.38,1.39]	0.60 [0.31,0.95]	0.66 [0.54,0.77]	0.58 [0.45,0.70]	0.62 [0.47,0.75]	0.69 [0.54,0.84]
13	0.75 [0.57,0.93]	0.66 [0.25,0.99]	0.57 [0.34,0.84]	0.90 [0.43,1.42]	0.69 [0.58,0.80]	0.72 [0.52,0.92]	0.58 [0.50,0.66]	0.71 [0.48,0.95]

For each subject mean α is shown on the first line and the 95% CI on the second line.

To further evaluate robustness, we performed a sensitivity analysis by varying *k* around the selected value (*k*±2). The results (Tables 5–8 in Supplementary File 3) show that the computed scaling exponents (α) remain stable across all subjects, ragas, and approaches (norm-based and mean-based). The mean values of the scaling exponent (α) vary marginally (typically within ~0.01–0.03) across different *k* values (*k*−2 to *k*+2), while the corresponding standard deviations also show no systematic trend. These findings indicate that the proposed approach is not highly sensitive within the examined range of *k*, thereby supporting the robustness of the adaptive neighborhood selection.

The segmentation of the EEG data into 2-s windows yields approximately *n*≈90 windows, which limits the scale range for DFA (*p*∈[4, *n*/4]). This may influence the robustness and precision of the estimated scaling exponent (α). Although consistent linear trends were observed in the log–log plots, the mean-based approach exhibited comparatively more stable estimates than the norm-based approach. Furthermore, bootstrap analysis indicated that the estimated α values were generally consistent with their corresponding confidence intervals, supporting the stability of the results within the available scale range. Since the same methodology was applied uniformly across all subjects and conditions, the comparative differences reported in this study remain meaningful. Future work incorporating longer recordings would enable a broader scale range and potentially improve the reliability of scaling exponent estimation.

### 5.1 Application of the proposed framework on Music BCI dataset (006-2015)

To evaluate the proposed algorithm, we used the Music BCI (006-2015) dataset, which was selected for its relevance to the musical paradigm under investigation. This dataset, originally introduced by Treder et al. (2014), contains EEG recordings from eleven volunteers and was deemed suitable for assessing our approach. A 64-channel EEG cap with an international 10–10 electrode placement system was used. The authors utilized various musical instruments to generate Jazz and Synth-Pop musical clips. There was no relaxation period in the dataset, so we have applied our framework only to the musical clips. According to Treder et al. (2014), the EEG data were initially collected at a sampling frequency of 1,000 Hz and subsequently downsampled to 200 Hz, and artifacts were removed using a min-max criterion. We have applied the same preprocessing steps as used for the first dataset to maintain consistency across analyses. The EEG data were z-score normalized, band-pass filtered in the alpha-band (8–13 Hz), segmented into non-overlapping 2-s windows, and the alpha-band power was extracted for each segment. This ensures that both datasets are processed using a consistent pipeline, enabling a fair comparison of the results.

**Figure 7:**
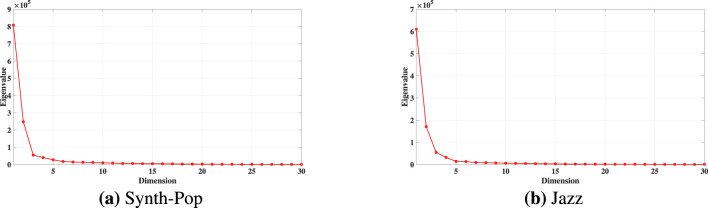
Eigenvalue spectra of BCI dataset using Isomap on Subject VPjaq. **(a)** Synth pop, **(b)** Jazz. The elbow after the fifth eigenvalue indicates an optimal embedding dimension of *d* = 5.

**Figure 8:**
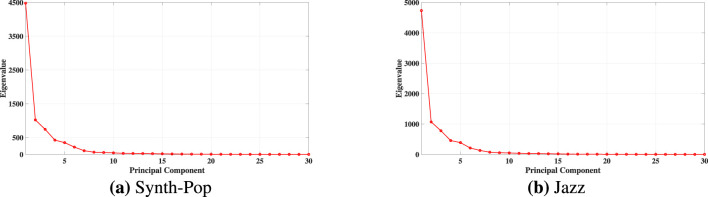
Eigenvalue spectra after performing PCA for Subject VPaaq on the BCI dataset. **(a)** Synth pop, **(b)** Jazz. The elbow after the eighth eigenvalue indicates an optimal embedding dimension of *d* = 8.

The eigenvalue spectra for both musical clips obtained using Isomap and PCA are shown in Figures 7, 8, respectively. A clear elbow is observed after the fifth dimension in the Isomap subplots (Figures 7a, b). Consistent patterns in the eigenvalue decay were observed across all subjects, with a clear elbow occurring at *d* = 5. Based on this observation, five (5) dimensions were selected for the reduced Isomap embedding. In contrast, inspection of the PCA eigenvalue spectra (Figures 8a, b) indicated that eight (8) dimensions were required. Similar to the ICM dataset, the BCI dataset also required more dimensions when using PCA compared to Isomap.

The mean values of scaling exponent (α) and goodness-of-fit (*R*^2^) across all subjects, along with their standard deviations, are presented in Table 10. For both methods (Isomap and PCA) and approaches (norm-based and mean-based), the α values are within the range of LRTCs. Similar to the ICM dataset, for some subjects, the α values for the norm-based approach in both methods are less than 0.5 and greater than 1. However, the mean-based approach produced more stable estimates of the scaling exponent (α), with values consistently within the expected range of LRTC (0.5 < α < 1) and higher goodness-of-fit values (refer Tables 1–4 of Supplementary File 3). This consistency with the BCI dataset demonstrates the robustness of the algorithm.

**Table 10:** Scaling exponent (α) and goodness of fit (*R*^2^) values (Mean ± Std) for both the music clips Synth-Pop and Jazz using both approaches and methods (Isomap and PCA).

		Synth Pop		Jazz	
Method	Approach	α	*R* ^2^	α	*R* ^2^
Isomap	Mean-based	0.65 ± 0.05	0.93 ± 0.03	0.62 ± 0.05	0.93 ± 0.03
	Norm-based	0.64 ± 0.26	0.90 ± 0.07	0.64 ± 0.16	0.86 ± 0.13
PCA	Mean-based	0.62 ± 0.03	0.92 ± 0.03	0.62 ± 0.04	0.92 ± 0.02
	Norm-based	0.63 ± 0.28	0.88 ± 0.10	0.65 ± 0.18	0.84 ± 0.17

A paired *t*-test comparing the Isomap mean-based and PCA mean-based approaches showed no statistically significant differences for both music clips, Synth-pop (*p* = 0.09, Bonferroni-corrected *p* = 0.18) and Jazz (*p* = 0.75, Bonferroni-corrected *p* = 1.00). Effect size analysis indicated a moderate effect for Synth-pop (Cohen's *d* = 0.57) and a negligible effect for Jazz (Cohen's *d* = 0.10). These results suggest a trend toward improved performance for Synth-pop, although statistical significance was not achieved, likely because of limited sample size.

## 6 Conclusions

In this study, we proposed a novel framework which combines nonlinear manifold learning (Isomap) with detrended fluctuation analysis (DFA) to characterize long-range temporal correlations (LRTC) in EEG recorded while individuals listened to live music. We also explored two approaches for applying DFA to low-dimensional embedding: norm-based and mean-based. Later, we compared it with an equivalent linear pipeline using PCA.

Across embeddings and approaches, the majority of scaling exponents (α) were in the interval 0.5 < α < 1, indicating persistent LRTC in the alpha band of EEG during both music and relaxation conditions. The nonlinear Isomap embedding captured the data structure with fewer dimensions (*d* = 3) than PCA (*d* = 5), suggesting that the EEG dynamics are represented on a low-dimensional nonlinear manifold. Mean α values estimated from nonlinear Isomap (norm-based approach α = 0.66–0.82; mean-based approach α = 0.66–0.70) and from linear PCA (norm-based approach α = 0.65–0.80; mean-based approach α = 0.62–0.67) consistently supported persistent scaling, while the mean-based DFA produced higher goodness-of-fit (*R*^2^) and more stable estimates (bootstrap CIs) than the norm-based approach. In particular, the relaxation condition showed larger α values than the music condition, and also the Raga Puriya Dhanashree produced higher mean α values than Raga Yaman. The Paired *t*-tests indicated that the Isomap framework of mean-based DFA was more sensitive than PCA- mean-based DFA to music-related changes (Yaman: *p* = 0.02; Puriya Dhanashree: *p* = 0.008). Comparative results were obtained on the Music BCI dataset using Isomap and PCA with embedding dimensions of 5 and 8, respectively. The scaling exponent (α) values for the mean-based approach fall within the range of 0.5–1.0 for both methods (Isomap and PCA) and for both Synth Pop and Jazz clips. These findings demonstrate the robustness and generalizability of the proposed algorithm.

In summary, the present study establishes a methodological framework that integrates nonlinear manifold learning with fractal analysis to uncover scale-free dynamics in brain activity. The consistent presence of persistent LRTC in EEG, along with the improved sensitivity of Isomap-based embeddings, demonstrates the utility of nonlinear approaches in studying complex physiological systems. Beyond the specific context of music perception, the proposed framework contributes to the broader understanding of fractal organization and nonlinear scaling laws in biological signals. In the future, the framework could be extended to other dimensionality reduction techniques to further assess generalizability. Moreover, as the current study focused on healthy individuals, extending the study to clinical populations would help determine the applicability of the method in translational neuroscience.

## Data Availability

The MATLAB (.p codes and EEG data for one participant) used in this work is available at our GitHub link below: https://github.com/NeuralLabIITGuwahati/LRTC_MusicEEG. Data for all participants in this study can be shared on request.

## Appendix

In the mean-based approach, the low-dimensional embedding *Y*∈ℝ^*n*×*d*^ is not reduced to a single time series. Instead, detrended fluctuation analysis (DFA) is applied independently to each dimension *Y*_*j*_, where *j* = 1, 2, …, *d*. This yields a set of scaling exponents α_*j*_, one for each dimension. The final scaling exponent is then obtained by averaging across all dimensions, as defined below. The detailed computation of the integrated series, fluctuation function, and scaling exponent is given in Equations 14–19.

$$\begin{array}{ccc} {Ȳ}_{j} & = & \frac{1}{n}\overset{n}{\underset{i=1}{\sum }}Y_{j}\left(i\right),\;j=1,2,\ldots ,d. \end{array}$$
(14)

$$Z_{j}(k)\;=\sum_{i=1}^{k}(Y_{j}(i)-{\bar{Y}}_{j}),$$
(15)

$$\begin{array}{ccc} k & = & 1,2,\ldots ,n,j=1,2,\ldots ,d. \end{array}$$
(16)

$$\begin{array}{ccc} p & \in & \left[p_{\min},p_{\max}\right],\;p_{\min}=4,p_{\max}=\left\lfloor \frac{n}{4}\right\rfloor . \end{array}$$
(17)

$$F_{j}(p)\;=\sqrt{\frac{1}{n}\sum_{k=1}^{n}(Z_{j}(k)-Z_{j,p}(k){)}^{2}},\;j=1,2,\ldots ,d.$$
(18)

$$\begin{array}{ccc} F_{j}\left(p\right) & \propto & p^{\alpha_{j}},\;j=1,2,\ldots ,d. \end{array}$$
(19)

$$\begin{array}{ccc} \alpha_{\text{mean}} & = & \frac{1}{d}\overset{d}{\underset{j=1}{\sum }}\alpha_{j}. \end{array}$$
(20)
