# Supplementary material for: Long-range correlations in alpha-band of electroencephalogram: a nonlinear embedding and detrended fluctuation analysis
Source: Front Neuroinform. 2026 May 20;20:1823408. doi: 10.3389/fninf.2026.1823408 (PMC13229972; doi:10.3389/fninf.2026.1823408)
Supplement: Supplementary file 2 [file Supplementary_file_2.docx]

**Eigenvalue spectra-Isomap**

**Yaman Puriya Dhanashree**

**Subjects**

1

2

3

4

5

6

7

8

9

10

11

13
