# Supplementary material for: Long-range correlations in alpha-band of electroencephalogram: a nonlinear embedding and detrended fluctuation analysis
Source: Front Neuroinform. 2026 May 20;20:1823408. doi: 10.3389/fninf.2026.1823408 (PMC13229972; doi:10.3389/fninf.2026.1823408)
Supplement: Supplementary file 3 [file Supplementary_file_3.pdf]

Table 1. Scaling exponent (α) and goodness-of-fit (R^2) for both musical clips Synth-Pop and Jazz using **mean-based** approach (**Isomap**).

**Subjects**	**Synth-Pop**		**Jazz**	
	**α**	**R^2**	**α**	**R^2**
VPjat	0.7324	0.9350	0.6710	0.9355
VPjaq	0.5779	0.9348	0.6062	0.9482
VPgeo	0.6491	0.9460	0.6059	0.9319
VPgcc	0.6477	0.9314	0.5619	0.9486
VPaat	0.6426	0.9433	0.6120	0.9556
VPaas	0.7406	0.9171	0.6150	0.8599
VPaar	0.6302	0.9387	0.6038	0.9551
VPaaq	0.6456	0.8785	0.6484	0.9348
VPaap	0.6888	0.8604	0.7272	0.9003
VPaan	0.5768	0.9544	0.5509	0.9344
VPaak	0.5891	0.9450	0.6018	0.8929

Synth-Pop mean values: α: 0.65 ± 0.05; R^2: 0.93 ± 0.03
Jazz mean values: α: 0.62 ± 0.05; R^2: 0.93 ± 0.03

Table 2. Scaling exponent (α) and goodness-of-fit (R^2) for both musical clips Synth-Pop and Jazz using **mean-based** approach (**PCA**).

**Subjects**	**Synth-Pop**		**Jazz**	
	**α**	**R^2**	**α**	**R^2**
VPjat	0.6703	0.9239	0.6671	0.9237
VPjaq	0.5975	0.9270	0.5961	0.9362
VPgeo	0.5747	0.9264	0.6102	0.9267
VPgcc	0.6329	0.9438	0.6291	0.9328
VPaat	0.6190	0.9368	0.5737	0.9386
VPaas	0.6445	0.9282	0.6165	0.9063
VPaar	0.6281	0.9063	0.5991	0.9518
VPaaq	0.6296	0.8964	0.6610	0.9166
VPaap	0.6346	0.8621	0.6864	0.9052
VPaan	0.6290	0.9455	0.5621	0.9366
VPaak	0.5961	0.9374	0.5870	0.8685

Synth-Pop mean values: α: 0.62 ± 0.03; R^2: 0.92 ± 0.03
Jazz mean values: α: 0.62 ± 0.04; R^2: 0.92 ± 0.02

Table 3. Scaling exponent (α) and goodness-of-fit (R^2) for both musical clips Synth-Pop and Jazz using **norm-based** approach (**Isomap**).

**Subjects**	**Synth-Pop**		**Jazz**	
	**α**	**R^2**	**α**	**R^2**
VPjat	0.6393	0.7993	0.5454	0.5128

VPjaq	0.8581	0.9804	0.7813	0.9647
VPgeo	0.6387	0.9249	0.6425	0.9254
VPgcc	0.5478	0.9336	0.5129	0.9012
VPaat	0.4479	0.9798	0.8125	0.9408
VPaas	1.2931	0.9328	0.5267	0.9276
VPaar	0.5513	0.9571	0.6328	0.9367
VPaaq	0.3864	0.8144	0.5756	0.7951
VPaap	0.7094	0.7862	0.9719	0.8550
VPaan	0.4574	0.8866	0.4168	0.9246
VPaak	0.4552	0.8913	0.6307	0.8010

Synth-Pop mean values: α: 0.64 ± 0.26; R^2: 0.90 ± 0.07
Jazz mean values: α: 0.64 ± 0.16; R^2: 0.86 ± 0.13

Table 4. Scaling exponent (α) and goodness-of-fit (R^2) for both musical clips Synth-Pop and Jazz using **norm-based** approach (**PCA**).

**Subjects**	**Synth-Pop**		**Jazz**	
	**α**	**R^2**	**α**	**R^2**
VPjat	0.5856	0.6697	0.4965	0.3931
VPjaq	0.8373	0.9775	0.8160	0.9667
VPgeo	0.6310	0.9224	0.6214	0.9016
VPgcc	0.5502	0.9293	0.5495	0.8877
VPaat	0.4151	0.9789	0.8210	0.9413
VPaas	1.3260	0.9263	0.5489	0.9190
VPaar	0.5167	0.9513	0.6185	0.9378
VPaaq	0.3392	0.7742	0.5234	0.7474
VPaap	0.7880	0.7557	1.0693	0.8121
VPaan	0.4025	0.8604	0.4387	0.9259
VPaak	0.4915	0.9065	0.6373	0.7610

Synth-Pop mean values: α: 0.63 ± 0.28 ; R^2: 0.88 ± 0.10
Jazz mean values: α: 0.65 ± 0.18 ; R^2: 0.84 ± 0.17

Table 5. Sensitivity analysis of the scaling exponent (α) for the **norm-based** DFA approach for Raga **Yaman** during and after music listening. Results are shown for neighborhood sizes (***k-2*** **to** ***k+2***).

**Subjects**	**During Music**					**After Music**				
	***k*-2**	***k* -1**	***k***	***k* +1**	***k* +2**	***k* -2**	***k* -1**	***k***	***k* +1**	***k* +2**
**1**	0.4928	0.4955	0.4815	0.4852	0.4919	0.4157	0.4083	0.4049	0.4021	0.4202
**2**	0.5496	0.5590	0.5534	0.5437	0.5648	0.6498	0.6491	0.6536	0.6503	0.6499

**3**	0.6234	0.6113	0.6075	0.6045	0.6060	0.4810	0.4967	0.4987	0.5026	0.5077
**4**	0.8326	0.8032	0.7800	0.7980	0.7894	0.9939	0.9923	0.9927	0.9855	0.9923
**5**	0.5455	0.5552	0.5439	0.5492	0.5482	0.8768	0.8705	0.8975	0.8812	0.8801
**6**	0.9626	0.9611	0.9537	0.9551	0.9615	0.8235	0.8421	0.8384	0.8441	0.8461
**7**	0.7463	0.7592	0.7641	0.7618	0.7660	0.7689	0.7537	0.7038	0.7208	0.7125
**8**	0.4546	0.4481	0.4457	0.4651	0.4742	0.6805	0.6727	0.6589	0.6651	0.6619
**9**	0.4304	0.4474	0.4261	0.4392	0.4303	0.7837	0.7684	0.8017	0.7718	0.7827
**10**	0.6914	0.7088	0.7214	0.6990	0.6685	0.4725	0.4526	0.4316	0.4237	0.4185
**11**	0.7736	0.7595	0.7782	0.7825	0.7833	0.7143	0.6913	0.6621	0.6501	0.6498
**12**	0.6753	0.7058	0.7104	0.7009	0.7005	0.6374	0.5984	0.6354	0.5929	0.5952
**13**	0.8177	0.8230	0.8341	0.8001	0.8348	0.8645	0.8423	0.8586	0.9128	0.8528
**Mean ± Std**	**0.66 ± 0.16**	**0.66 ± 0.15**	**0.66 ± 0.16**	**0.66 ± 0.15**	**0.66 ± 0.15**	**0.70 ± 0.17**	**0.69 ± 0.17**	**0.69 ± 0.17**	**0.69 ± 0.18**	**0.69 ± 0.17**

Table 6. Sensitivity analysis of the scaling exponent (α) for the **norm-based** DFA approach for Raga **Puriya Dhanashree** during and after music listening. Results are shown for neighborhood sizes (***k-2*** **to** ***k+2***).

**Subjects**	**During Music**					**After Music**				
	***k*-2**	***k* -1**	***k***	***k* +1**	***k* +2**	***k* -2**	***k* -1**	***k***	***k* +1**	***k* +2**
**1**	0.6266	0.6020	0.5907	0.5760	0.5647	1.6241	1.6245	1.6612	1.6384	1.6418
**2**	0.7119	0.7146	0.7167	0.7091	0.7267	0.5126	0.5274	0.5391	0.5420	0.5457
**3**	1.3224	1.3180	1.3191	1.3216	1.3252	0.4821	0.4822	0.4823	0.4869	0.4794
**4**	0.7951	0.7964	0.7465	0.7425	0.7162	0.6088	0.6234	0.6246	0.6262	0.6331
**5**	0.5366	0.5396	0.5386	0.5353	0.5370	0.6531	0.6209	0.6287	0.6213	0.6040
**6**	0.4054	0.3835	0.3972	0.4068	0.4123	0.9614	1.0036	1.0069	1.0078	0.9813
**7**	0.5919	0.5042	0.5335	0.5554	0.5731	0.5391	0.5232	0.5248	0.5120	0.5190
**8**	0.5641	0.5434	0.5496	0.5602	0.5526	0.8177	0.8612	0.8646	0.8538	0.8437
**9**	0.7737	0.7789	0.7913	0.8112	0.8022	0.6329	0.6218	0.6025	0.5637	0.5282
**10**	0.4708	0.5025	0.4527	0.4374	0.4428	0.6848	0.7004	0.7137	0.7265	0.6779
**11**	0.5994	0.6005	0.5977	0.5766	0.5794	0.9632	0.9459	0.9596	0.9614	0.9621
**12**	1.2769	1.2803	1.2890	1.2934	1.2930	0.6645	0.6778	0.6497	0.6906	0.6951

**13**	0.6297	0.6256	0.6269	0.6358	0.6418	1.5365	1.4661	1.4282	1.4335	1.4446
**Mean ± Std**	**0.71 ± 0.28**	**0.70 ± 0.28**	**0.70 ± 0.28**	**0.70 ± 0.29**	**0.70 ± 0.28**	**0.82 ± 0.36**	**0.82 ± 0.36**	**0.82 ± 0.36**	**0.82 ± 0.35**	**0.81 ± 0.36**

Table 7. Sensitivity analysis of the scaling exponent (α) for the **mean-based** DFA approach for Raga **Yaman** during and after music listening. Results are shown for neighborhood sizes (***k-2* to *k+2***).

**Subjects**	**During Music**					**After Music**				
	***k*-2**	***k* -1**	***k***	***k* +1**	***k* +2**	***k* -2**	***k* -1**	***k***	***k* +1**	***k* +2**
**1**	0.7621	0.7435	0.7418	0.7585	0.7579	0.6233	0.6300	0.6234	0.6245	0.6068
**2**	0.5227	0.4989	0.5238	0.5010	0.4931	0.7310	0.7729	0.7956	0.8104	0.7279
**3**	0.6218	0.6255	0.6246	0.6167	0.6228	0.5344	0.4985	0.5058	0.5114	0.5272
**4**	0.7495	0.7707	0.7889	0.7496	0.7032	0.8313	0.8474	0.8486	0.8407	0.8334
**5**	0.5655	0.5791	0.5726	0.5735	0.5780	0.5901	0.5181	0.6075	0.6156	0.5539
**6**	0.6390	0.6391	0.6280	0.6162	0.5971	0.6838	0.6965	0.6658	0.6702	0.6555
**7**	0.7595	0.7923	0.7933	0.7857	0.7705	0.7046	0.6997	0.7471	0.7375	0.7267
**8**	0.5024	0.5053	0.5103	0.4962	0.5103	0.8923	0.8851	0.9510	0.8635	0.8475
**9**	0.4986	0.4975	0.4851	0.4986	0.4853	0.6637	0.6573	0.6371	0.6437	0.6218
**10**	0.6780	0.6751	0.6771	0.6755	0.6675	0.7066	0.7594	0.7421	0.6823	0.7108
**11**	0.7946	0.8042	0.8023	0.8097	0.8176	0.6223	0.6210	0.6330	0.6362	0.6265
**12**	0.7098	0.7144	0.7331	0.7252	0.7313	0.5931	0.5202	0.5692	0.6036	0.5674
**13**	0.6618	0.7361	0.7200	0.7145	0.7084	0.8371	0.7578	0.8337	0.8768	0.7660
**Mean ± Std**	**0.65 ± 0.10**	**0.66 ± 0.11**	**0.66 ± 0.11**	**0.65 ± 0.11**	**0.64 ± 0.11**	**0.69 ± 0.10**	**0.68 ± 0.12**	**0.70 ± 0.12**	**0.70 ± 0.11**	**0.67 ± 0.10**

Table 8. Sensitivity analysis of the scaling exponent (α) for the **mean-based** DFA approach for Raga **Puriya Dhanashree** during and after music listening. Results are shown for neighborhood sizes (***k-2* to *k+2***).

**Subjects**	**During Music**					**After Music**				
	***k*-2**	***k* -1**	***k***	***k* +1**	***k* +2**	***k* -2**	***k* -1**	***k***	***k* +1**	***k* +2**
**1**	0.6059	0.5849	0.6063	0.5962	0.6064	0.9377	0.8625	0.8949	0.8972	0.9038
**2**	0.6749	0.6566	0.7145	0.6989	0.6721	0.5248	0.5065	0.6096	0.6331	0.6349

**3**	0.7404	0.7403	0.7563	0.7453	0.7497	0.5658	0.5585	0.5365	0.5631	0.6008
**4**	0.5491	0.5565	0.5455	0.5498	0.5204	0.6086	0.5763	0.5632	0.5648	0.5753
**5**	0.6293	0.6250	0.6226	0.5981	0.5828	0.6605	0.6404	0.5764	0.5576	0.5696
**6**	0.6044	0.6294	0.5848	0.5901	0.6021	1.0746	0.8486	0.7616	0.6901	0.6699
**7**	0.6867	0.8400	0.8097	0.7877	0.7520	0.6365	0.6072	0.6226	0.6153	0.6189
**8**	0.5726	0.5748	0.5739	0.5780	0.5667	0.8743	0.8941	0.8865	0.8130	0.8081
**9**	0.6882	0.7199	0.7512	0.7734	0.7537	0.6416	0.6655	0.6477	0.6866	0.6212
**10**	0.8412	0.8304	0.8430	0.8420	0.8384	0.6008	0.6401	0.6275	0.6510	0.6060
**11**	0.6588	0.6696	0.6605	0.6559	0.6554	0.6856	0.6584	0.6731	0.6689	0.6422
**12**	0.8999	0.9095	0.9087	0.8911	0.9185	0.7566	0.7788	0.7760	0.7877	0.7847
**13**	0.5844	0.5773	0.5766	0.5848	0.5808	0.8639	0.8760	0.8678	0.8251	0.8832
**Mean ± Std**	**0.67 ± 0.10**	**0.68 ± 0.11**	**0.68 ± 0.11**	**0.68 ± 0.11**	**0.67 ± 0.11**	**0.72 ± 0.16**	**0.70 ± 0.13**	**0.69 ± 0.12**	**0.68 ± 0.11**	**0.68 ± 0.11**

Table 9: Comparison of scaling exponent (α) (Mean ± Std) for Isomap and PCA at d = 5 for both the approaches.

Raga	Condition	Isomap (Norm)	Isomap (Mean)	PCA (Norm)	PCA (Mean)
**Yaman**	**During Music**	0.66 ± 0.15	0.61 ± 0.08	0.65 ± 0.16	0.62 ± 0.07
	**Relaxation**	0.69 ± 0.18	0.65 ± 0.08	0.71 ± 0.19	0.66 ± 0.05
**Puriya Dhanashree**	**During Music**	0.70 ± 0.28	0.62 ± 0.07	0.71 ± 0.29	0.63 ± 0.06
	**Relaxation**	0.81 ± 0.37	0.64 ± 0.08	0.80 ± 0.36	0.66 ± 0.07
